# A Comparison between Cold Dissection Tonsillectomy and Harmonic Scalpel Tonsillectomy

**Published:** 2017-11

**Authors:** Ebrahim Karimi, Alireza Safaee, Shahin Bastaninejad, Soheila Dabiran, Elham Masoumi, Farnaz Moravej Salehi

**Affiliations:** 1 *Otorhinolaryngology Research Center, Tehran University of Medical Sciences, Tehran, Iran.*; 2 *Department of Epidemiology, Community Medicine, Tehran University of Medical Sciences, Tehran, Iran* *.*

**Keywords:** Harmonic scalpel, Postoperative Hemorrhage, Tonsillectomy surgical methods, Tonsillectomy

## Section Title

### Introduction:

This study aimed to compare operation time, intraoperative bleeding and postoperative pain between cold dissection tonsillectomy and harmonic scalpel tonsillectomy.

### Materials and Methods:

In this single-blinded clinical trial, 32 patients aged 14–48 were enrolled. Each patient randomly underwent tonsillectomy using the harmonic scalpel on one side and cold dissection on the other side. Operation time and bleeding volume were measured during surgery. The pain intensity level on each side was recorded on the first and seventh postoperative days.

### Results:

The mean volume of intraoperative bleeding was 9.59 ml on the harmonic side and 74.38 ml on the cold dissection side; which represents a significantly lower amount on the harmonic side (P<0.001). The mean time of tonsillectomy was 427.63±196.32 s for the harmonic side and 711 271.88 s for the cold dissection side (P<0.001). The mean pain intensity level on the first postoperative day was 3.88 on the harmonic side and 6.19 on the cold dissection site (P<0.001).

### Conclusion:

Mean operation time, volume of bleeding and pain intensity level on the first postoperative day were all statistically lower on the harmonic side.

## Introduction

Tonsillectomy is one of the most common head and neck surgeries. Indications for tonsillectomy in adults include chronic or recurrent tonsillitis, airway obstruction due to hypertrophied tonsils or any other suspicious tumors of the tonsils (1).

Several surgical methods have been tested for the reduction of complications and improvement in surgical outcome. Until the 1960s, tonsillectomy was performed using the cold dissection method. Gradually with technological advances, other methods such as monopolar and bipolar electrocautery, CO_2_ laser, bipolar radiofrequency, harmonic scalpel and vessel sealing systems were added, all of which differ in terms of intraoperative bleeding, operation time, pain after surgery, time of starting oral nutrition and return to usual activities (2).

A harmonic scalpel was first used for tonsillectomy in the year 2000. In this method, the blade of the instrument vibrates at 55 kHz, such that it can cut tissues and coagulate vessels simultaneously (3,4).

Several studies have been performed which compare the complications of this new method with other common methods, and have reported different and paradoxical results. In many studies, intraoperative and postoperative bleeding rates with the harmonic method were lower than with cold dissection and electrocautery methods (5–7). However, in other studies different results were reported (2,8–12).

In this study, we compared different variables such as tonsillectomy time, intraoperative and postoperative bleeding and postoperative pain with both methods (harmonic scalpel and cold dissection) to select the best method according to the patient’s condition.

## Materials and Methods

The present single-blinded, randomized, clinical trial was conducted under registration number IRCT201609179039N4 and with the approval of the Ethics Committee of the Otorhinolaryngology Research Center of Tehran University of Medical Sciences. Determination of sample size was achieved by comparing two means formula, with available data from the study conducted by Oko et al. (10). The study population included 32 patients, 14–48 years of age, who were enrolled at Amir’Alam Hospital, a tertiary referral otolaryngology hospital in Tehran. These patients were candidates for tonsillectomy due to recurrent tonsillitis and tonsillar hypertrophy. Patients with coagulopathy, a history of previous similar surgery, lingual, mental and developmental problems and sleep apnea were excluded from the study.

**Fig 1:**
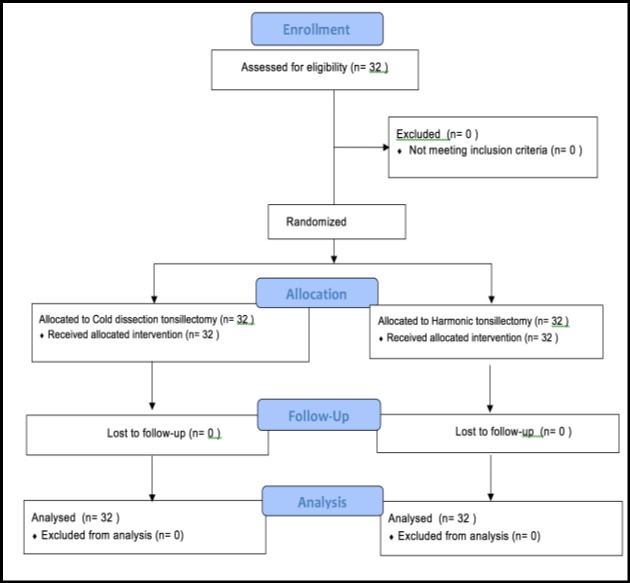
Consort follow diagram

Patients entered the study after receiving necessary explanations and giving consent to enter. All patients underwent surgery by the same surgeon with both harmonic scalpel and cold dissection techniques (Fig.1). 

The surgical procedure used on the patient’s right side was randomly assigned using a block randomization method. We designed blocks, containing 2× “H” letters (for harmonic) and 2× “C” letters (for cold dissection) in each block, making six possible arrangements (HHCC, HCHC, etc.). We then chose one of the blocks randomly (using a random numbers table) and assigned it to four patients. Repeating this eight times dictated the method of surgery on the right side for the 32 patients; the left tonsil was then approached using the other surgical technique. Bleeding on either side was stopped by means of a figure-of-eight suturing technique.

During surgery, time was calculated using a chronometer from incision to the end of coagulation. Intraoperative bleeding was calculated from the suction bottle (with syringe) and by weighing of blood gauzes. All patients received one dose of dexamethasone before surgery and therapeutic doses of acetaminophen after surgery. Postoperative pain was assessed using a numeric rating pain scale (from zero to 10: zero for no pain, 10 for the most severe pain) during the first and the seventh postoperative days.

Normal distribution of data was assessed using the Shapiro-Wilk test. Comparison of means was performed using the independent t-test (in normal distributed variables) and the Mann-Whitney U-test (in other variables). Qualitative variables were compared using the Fisher’s exact test and the Pearson chi-squared test. The correlation between variables was assessed using the Kendall’s Tau-b method, and the results were analyzed using the SPSS version 24.

## Results

In total, 32 patients (19 males; 59.4 % and 13 females; 40.6%) were included in the study, with a mean age of 27.4±7.9 years. With the harmonic method, the mean bleeding volume during surgery was 9.59 ml, whereas with the cold dissection method, it was 74.38 ml, with a significant difference (P<0.001) (Table.1).

**Table 1:** Mean and standard deviation variables for the two surgical methods

**Outcomes **	**Harmonic scalpel tonsillectomy** **Mean (SD)(n=32)**	**Cold dissection tonsillectomy** **Mean (SD) (n=32)**	**P-value**
Intraoperative bleeding (ml)	9.59 (16.7)	74.38 (271.8)	0/000*
Operation time (s)	427.3 (196.32)	747.84 (271.88)	0/000*
Pain: Day 1 (score)	3.88 (2.22)	6.19 (2.16)	NS*
Pain: Day 7 (score)	3.09 (2.22)	2.59 (1.91)	NS*

* 0/000: P<0.0001;

* NS: Non-significant

Tonsillectomy time from incision to hemostasis was 427.63±196.32 s with the harmonic method and 747.84±271.88 s with the cold dissection method, with a statistically significant difference (P<0.001).On the first and seventh days, pain intensity was recorded using a numeric rating pain scale. Mean pain severity on the first day after surgery was 3.88 on the harmonic side and 6.19 on the cold dissection side, with a significant difference (P<0.001). No significant difference was found between pain intensity on the seventh day after surgery between the harmonic (3.09) and cold dissection (2.59) methods (P=0.368).

No significant difference was detected in comparison of the patients' gender with bleeding rate during surgery and pain intensity on first and seventh days after surgery. Further, the bleeding rate after surgery was 6.25% with the harmonic method and 9.37% with the cold dissection method; thus, no significant difference was seen in this parameter either.

Using Kendall’s Tau-b test for correlation assay between variables, a positive correlation was found between pain on the first day after surgery and bleeding volume during surgery in the harmonic group (P=0.009; r=0.358). In the cold dissection group, no correlation between variables was detected.

Across both methods, the following positive correlations were detected: 1) Bleeding volume and duration of surgery (p<0.001; r=0.462); 2) Pain on the first day after surgery and bleeding volume (P<0.001; r=0.362); 3) Pain intensity on the first day after surgery with duration of surgery (P=0.003; r=0.269).

## Discussion

Tonsillectomy is one of the oldest and the most common head and neck surgeries. Despite its technical simplicity, there is considerable concern about dangerous and fatal complications such as bleeding. Many studies have been performed to assess the safest method in terms of intraoperative and postoperative complications.

In this study, the mean volume of bleeding during surgery in the harmonic group was 9.59 ml, and in the cold dissection method it was 74.38 ml, with a significant difference between the two methods. In previous studies, such as those by Akural in 2001, Oko in 2005, Lachanas in 2007 and Alexiou in 2011, this difference had also been significant (2,5,7,10).

Although in Walker's study and Morgenstein's study in 2002 and Leaper's study in 2006 there was no significant difference in bleeding rate during surgery between harmonic and electrocautery methods (3,11,13),Morgenstein's study was non-randomized with non-similar groups resulting in less accuracy in this study.

In our study, the mean time of surgery was significantly less in the harmonic group (P<0.001). In Lachanas's study, this mean time of surgery was also significant. However, unlike other studies, in our study the duration of surgery in each group was recorded exactly from incision time until hemostasis, and was then compared with the results of the other group, without any other conflicting factors such as adenoidectomy time, additional hemostasis or irrigation time.

In our study, the bleeding rate after surgery was 6.25% with the harmonic method and 9.37% with the cold dissection method; thus, no significant difference was seen. Other studies also reported no significant difference in postoperative bleeding between these two groups (2,7,10,11,14,15).

In this study, postoperative pain (on the first and seventh days) was recorded using a numeric rating pain scale (from zero to 10), with results that were significantly lower on the harmonic side on the first day (3.88 versus 6.19; P<0.001), but not on the seventh day (P=0.328). In studies conducted by Akural and Oko, the harmonic and cold dissection methods were compared, and postoperative pain results were similar to our study (5,10). In studies conducted by Leaper, Panavic and Kemal, postoperative pain in the harmonic group was greater than in the bipolar group (11,14). Studies conducted by Cushing and Alexiou found no significant differences in postoperative pain between harmonic and electrocautery methods (2,9). In pain assessment with quantitative scales, different variables such as age, gender, anxiety and different pain thresholds can affect the study. To minimize the effect of these conflicting factors, we operated using two methods for each patient, and selected adult patients for a more accurate assessment of pain intensity and localization.

## Conclusion

In conclusion, use of the harmonic scalpel method in tonsillectomy showed significantly better outcomes compared with conventional cold dissection methods in terms of operation time, intraoperative bleeding and postoperative pain on the first postoperative day; however, there was no statistically significant difference in other parameters such as late postoperative bleeding and pain on the seventh postoperative day. Our study was performed in adult patients (14–48 years), but complementary studies are needed to confirm use of this method as a standard protocol for tonsillectomy in all age groups and in comparison with other surgical modalities. However, despite the benefits of the harmonic scalpel technique, the higher cost of the harmonic system and its imitation in reusing the hand piece should be kept in mind.
